# Editor's Letter-Box

**Published:** 1897-03-27

**Authors:** George Stoker


### Editor's Letter-Box.

[Our Correspondents are reminded that prolixity is a great bar to publication, and that brevity of style and conciseness of statement greatly facilitate early insertion.]

Mr. George Stoker writes: I trust you will give me an opportunity of replying to the criticisms you have kindly published on the "Oxygen Treatment." If the general hospitals have failed to investigate this system because there are no cases of chronic ulcers in their wards (which I very much doubt) surely they had sufficient recent cases, burns, scalds, wounds, &c., to put it to a trial. With regard to the question of ethics I beg to point out that for two years I kept patients in nursing homes, &c., for the purpose of perfecting and teaching this system; that my means being exhausted and my field of observation limited, the Oxygen Home was started by an influential committee as a centre for teaching and developing the system, and I would ask, in view of the prolonged indifference shown by hospital surgeons, "What possible means had I of establishing this treatment or collecting evidence if the Oxygen Home had not been established?" With regard to the criticisms you publish it is gratifying to know that there are some gentlemen who have the courage of their opinions and are prepared to back them with their names. So far as one can judge, it appears that those who have seen the treatment in practice speak approvingly, or, at least, keep an open mind. The severest condemnations seem to come from those who have little or no experience of it. I see that some of your correspondents consider me "almost criminal," or guilty of "inexcusable ignorance." But is not this begging the whole question? These gentlemen seem to have set up a theory for themselves that certain micro-organisms must in all cases work evil. But that is exactly the point on which we differ. My clinical experience of how ulcers heal, and my bacteriological investigations into the nature of the micro-organisms which accompany the process, show me that they are wrong, and that under the conditions which prevail under oxygen these microbes have not that evil effect which has, on theoretical grounds, been attributed to them; and, surely, nothing can be more absurd than for these gentlemen to throw aspersions upon me, because I show the errors of their assumptions, at the very time they themselves refuse to try the experiments which would reveal to them the truth. By all means let the system be tested, both clinically and bacteriologically; but I respectfully protest against any trials being accepted as conclusive unless they are conducted by those (both surgeons and nurses) who have a practical knowledge and experience of the working and details of the system, and on fairly selected cases.

